# Application of BRAFO-Tiered Approach for Risk–Benefit Assessment of Nut Consumption in Chinese Adults

**DOI:** 10.3390/foods14203498

**Published:** 2025-10-14

**Authors:** Zhujun Liu, Xiangyu Bian, Yingzi Zhao, Jiang Liang, Lei Zhang, Pingping Zhou, Weifeng Mao, Depeng Jiang, Pei Cao, Jinfang Sun

**Affiliations:** 1 Department of Epidemiology and Biostatistics, School of Public Health, Southeast University, Nanjing 210009, China; zhujun_liu@seu.edu.cn (Z.L.);; 2 China National Center for Food Safety Risk Assessment, Beijing 100022, China; 3 Key Laboratory of Environmental Medicine Engineering, Ministry of Education, School of Public Health, Southeast University, Nanjing 210009, China; 4 Department of Community Health Sciences, University of Manitoba, 7750 Bannatyne Ave, Winnipeg, MB R3T 2N2, Canada

**Keywords:** nuts, risk–benefit assessment, BRAFO, liver cancer, coronary heart disease, aflatoxins, Disability-Adjusted Life Year

## Abstract

Nuts are nutrient-rich foods that help reduce the risk of coronary heart disease (CHD), but their potential contamination with aflatoxins (AFs) may increase the risk of liver cancer. In this study, the European Benefit–Risk Analysis for Foods (BRAFO) framework was used to evaluate both the health risks and benefits of nut consumption among Chinese adults. Based on the actual consumption patterns of nuts among the Chinese population, the current consumption level was set as the reference scenario (4.66 g/day), and three alternative scenarios were simulated with a daily nut consumption of 10, 20, and 30 g, respectively. Dose–response relationships were established using a two-stage dose–response analysis for nut consumption and CHD risk, and a one-stage dose–response analysis for aflatoxin B1 (AFB1) exposure and liver cancer risk. A Monte Carlo probabilistic model quantified the CHD prevention benefits and liver cancer risks associated with AF exposure. Disability-Adjusted Life Year (DALY) analysis indicated net health benefits in all scenarios, with nut consumptions of 10, 20, and 30 g/day reducing DALYs per 100,000 population by 104.39, 143.63, and 181.47 in men, and by 58.79, 81.29, and 102.94 in women, respectively. A nut consumption of 10 g/day was recommended for Chinese adults, considering both health benefits and the risk of AF exposure. This study presents the first application of the BRAFO framework to evaluate the net health effect of nut consumption in a Chinese population, filling a critical gap in the risk–benefit assessment of nut consumption.

## 1. Introduction

Nuts are a category of nutritious foods with unique flavors, rich in protein, fats, vitamins, minerals, and dietary fiber. Numerous studies have indicated that nuts offer a variety of health benefits and effects, including the reduction of blood lipids and antioxidant prevention of cardiovascular disease [1,2]. Some prospective cohort studies have shown that higher nut consumption is linked to a lower risk of cardiovascular diseases (CVDs), with a 19% reduction in CVD incidence and a 25% decrease in mortality [3]. The American Heart Association and the European Society of Cardiology have suggested that nuts should be consumed regularly to lower LDL cholesterol, improve overall lipoprotein levels, and reduce the risk of CVD [4,5]. In China, *the Dietary Guidelines for Chinese Residents* (2022) recommend a weekly intake of 70 g of nuts, which is considered beneficial to health [6]. Although nuts are known to be rich in various healthy nutrients, they are prone to be contaminated with harmful substances like aflatoxins (AFs) [7], which may pose potential health risks to consumers [8].

AFs are a group of toxic secondary metabolites produced mainly by *Aspergillus flavus* and *Aspergillus parasiticus*. More than 18 types of AFs are known, of which aflatoxin B1 (AFB1), aflatoxin B2 (AFB2), aflatoxin G1 (AFG1) and aflatoxin G2 (AFG2) are the most extensively studied and widely reported [9]. The International Agency for Research on Cancer (IARC) has classified natural AFs as group 1 carcinogens [10]. Meanwhile, concurrent infection with hepatitis B virus (HBV) amplifies the risk of hepatocellular carcinoma (HCC) caused by AFs [11]. A wide variety of food crops, including cereals, legumes, oilseeds, and nuts, particularly peanuts, are susceptible to contamination by AFs [12]. A study on AF contamination and exposure in peanuts and peanut oil across China indicated that consumers in some southern provinces of China may have potential health risks [12]. To sum up, although nuts are widely recognized for their nutritional benefits, close attention should be paid to the potential health risks raised by contamination with AFs, highlighting the importance of risk–benefit assessment (RBA).

RBA is a comprehensive evaluation of the health impact of consuming a food or food component at a given exposure level, through the characterization of its potential risks and benefits [13]. RBA has been extensively applied to assess the health effects of various food categories, including fish and cereals [14,15]. BRAFO is a stepped RBA approach to quantitatively assess and compare the potential risks and benefits of foods using harmonized metrics [13]. This method has primarily focused on foods including fish [16], thermally processed foods [17], coffee [18], and nutrients (nitrates) [19]. In China, this approach is still in its developmental stage, with limited research primarily focused on marine fish [20] and black tea [21].

In this study, a Benefit–Risk Analysis for Foods (BRAFO) approach was used to assess nut consumption in China, with both risks and benefits quantified in the same health metric, the Disability-Adjusted Life Year (DALY). In addition, a dose–response relationship between nut consumption and the incidence of coronary heart disease (CHD) and the relationship between AFB1 exposure and the incidence of liver cancer were established, to assess the health effects more accurately. This study addressed the gap in RBA studies on nuts in China, and provides valuable insights for risk management and reasonable consumption recommendation.

## 2. Material and Methods

### 2.1. Concentration Data of AFs in Nuts

The concentration data of AFs(AFB1, AFB2, AFG1, and AFG2) in nuts were obtained from the China National Food Safety Monitoring Program in 2013 [22], which was comprehensive and systematically implemented. A total of 3349 samples were collected from supermarkets and local markets in China, covering 15 types of nuts. The analysis of AF levels was carried out using high-performance liquid chromatography (HPLC), in accordance with the China National Food Safety Standards GB/T 5009.23-2006 [23]. For analyses following GB/T 5009.23-2006, samples were extracted with acetonitrile–water, purified through multifunctional purification columns, evaporated under nitrogen, and derivatized using hexane and trifluoroacetic acid. After incubation and drying, the extract was redissolved in a water–acetonitrile mixture and centrifuged. The supernatant was analyzed by HPLC with a fluorescence detector (excitation 360 nm, emission 440 nm) using a C18 column with an acetonitrile–water mobile phase. The limits of detection (LOD) of AFB1, AFB2, AFG1 and AFG2 required in GB/T 5009.23-2006 were 0.20, 0.05, 0.20, 0.05 μg/kg, respectively. To guarantee data consistency and reliability, the laboratories underwent certification by the China National Safety Center for Food Risk Assessment for their detection-process quality control.

Table 1 presents the sample size, detection rate, mean, 95th percentile, and maximum concentration of total AFs for different types of nuts. Sunflower seeds showed the highest detection rate and relatively high mean concentrations among positive samples, whereas no AFs were detected in chestnuts or pecans. Therefore, this study specifically evaluated the potential health effects of increasing the consumption of sunflower seeds or pecans, which were selected to represent high-contaminated and non-contaminated nuts, respectively, in alternative scenarios.

### 2.2. Nut Consumption Data

The nut consumption data were obtained from the China National Food Consumption Survey (2018–2020) using the 24 h recall method over 3 discontinuous days, including one weekend day (Saturday or Sunday) and two weekdays. Food consumption was recorded through face-to-face interviews, and two adjacent surveys of the three interviews were conducted at least five days apart. This survey used a multistage random cluster sampling approach and was carried out across 18 provinces in China [24]. A total of 55,700 individuals participated in the survey, including 7121 nut consumers aged 18–94 years, of whom 3332 were men and 3789 were women. The average consumption of nuts by nut consumers was 4.66 g/day, among which peanuts, sunflower seeds, chestnuts and walnuts were the top 4 nuts in terms of average daily intake among the Chinese consumers.

### 2.3. Risk–Benefit Assessment

The BRAFO-tiered approach systematically evaluates the health risks and benefits associated with transitioning from the reference scenario to an alternative scenario, ultimately identifying which scenario is more favorable in terms of overall health impact. It typically consisted of five steps: (1) pre-assessment and question formulation; (2) individual assessment of risk and benefit (Tier 1); (3) qualitative integration of risk and benefit (Tier 2); (4) deterministic calculation of the common metric (Tier 3); and (5) probabilistic calculation of the common metric (Tier 4) [25].

#### 2.3.1. Pre-Assessment and Question Formulation

The scope of RBA was defined by identifying the food of interest, target population, benefit–risk considerations, and relevant dietary scenarios [25]. This study focused on nuts. The RBA compared a reference scenario, based on observed consumption levels, with a series of alternative scenarios defined by target consumption levels. In each alternative scenario, individuals with intake below the target were assumed to increase their consumption accordingly, while those exceeding the target maintained their current consumption [26]. This RBA studied nut consumption among Chinese adults aged 18 to 101 years old. According to *the Dietary Guidelines for Chinese Residents* (2022) [6], a weekly consumption of approximately 70 g (about 10 g/day) of nuts was advised, whereas Australian and American guidelines suggest 30 g/day for adults [27]. Therefore, three alternative scenarios for nut consumption were established at 10, 20, and 30 g/day, with the current adult nut consumption serving as the reference scenario.

#### 2.3.2. Tier 1

The objective of Tier 1 was to identify all potential health outcomes associated with transitioning from the reference scenario to the alternative scenario. Evidence on the beneficial effects of nuts and adverse effects of AFs exposure was based on human and/or animal studies. Literature quality was evaluated using established methods, and evidence strength was categorized as “convincing”, “probable”, “possible”, or “insufficient” [13]. To minimize subjectivity, a dual-review approach was adopted, with “convincing” evidence prioritized for further assessment.

#### 2.3.3. Tier 2

In this Tier, diseases supported by convincing evidence were identified for further analysis. The assessment of risks and benefits was informed by available data concerning both the severity of outcomes and the estimated number of individuals impacted. The analysis integrated various dimensions of health impacts, including morbidity, severity expressed as disability weights (ω), disease-specific mortality rates, and current DALYs, to qualitatively assess the balance of adverse and beneficial effects. When these dimensions were insufficient to determine a clear outcome, quantitative assessment proceeded to Tier 3 [21].

#### 2.3.4. Tier 3

In Tier 3, the DALY was employed as the standardized metric for quantifying each health impact assessed in this study. Directly attributable health impacts were quantified by calculating DALYs based on exposures to adverse and beneficial substances, dose–response relationships, and disease incidence across dietary scenarios.

##### Estimation of Dietary Exposure to Total AFs from Nuts in Chinese Adults

The probabilistic assessment modeling employed in this study was based on Monte Carlo simulations, which simulated the daily dietary exposure by sampling from the consumption database and combining these data with a random sample from the distribution of total AF concentrations in each type of nut. The random sampling from the concentration distribution was performed according to the percentage of the sample with detectable total AF concentrations and the percentage of the sample with undetectable total AF concentrations. The number of Monte Carlo iterations was 100,000 and the number of simulations was 100. The dietary exposure to total AFs was calculated according to the following formula [28]:

(1) $$y_{ij}=\frac{\sum_{k}^{p}x_{ijk}\times c_{ij}}{w_{i}}$$

where $y_{ij}$ was the exposure to total AFs from nuts by individual i on day  j (ng/kg bw day); $x_{ijk}$ was the consumption of the kth food by individual i on day j (g/day); $c_{ij}$ was the total AF concentration in the kth food by individual i on day j (μg/kg); p was the number of food types consumed by individual i; and $w_{i}$, was individual i body weight (kg).

Due to insufficient in vivo data, AFB2, AFG1, and AFG2 were assumed to have the same carcinogenic potency as AFB1. Total AF concentrations were calculated following EFSA guidelines, using the sum of detected values and upper-bound estimates for non-detects, depending on the detection status of each compound [29].

Concentration data were fitted to four candidate distributions (Gamma, lognormal, exponential, and Weibull), with the best fit selected based on Kolmogorov–Smirnov and Anderson–Darling tests. To ensure the representativeness and stability of the fitted distribution parameters, nuts with more than 10 positive detections were fitted individually [30]. For nuts with fewer than 10 positive detections, the total AF concentration data were pooled by classification into tree nuts or seed nuts before fitting.

##### Dose–Response Relationship

Search Strategy

PubMed, Web of Science, and CNKI were searched for relevant literature published up to February 2025. For studies on nut consumption and the risk of CHD, the search terms included “nuts” combined with “coronary heart disease,” “coronary artery disease,” “cardiovascular disease,” “myocardial infarction,” “ischemic heart disease,” “CHD,” or “IHD.” For studies on AFB1 exposure and the risk of liver cancer, the terms included “aflatoxin” or “AFB1” combined with “liver cancer,” “chronic liver disease,” “hepatocellular carcinoma,” or “HCC.” Both English- and Chinese-language publications were considered.

Inclusion and Exclusion Criteria

Inclusion criteria for studies for nut consumption and the risk of CHD: (i) observational studies in humans with cohort design; (ii) nuts as an exposure factor; (iii) outcomes of CHD reported; (iv) data provided of exposure levels, adjusted relative risk (RR) and 95% confidence interval (CI). Inclusion criteria for studies of AFB1 exposure and the risk of liver cancer: (i) case-control study design; (ii) AFB1 as the exposure of interest; (iii) liver cancer as the outcome of interest; (iv) reported odds ratios (ORs) with 95% CIs.

Conversely, studies that met the following criteria were excluded: (i) publications such as review, editorial, commentary, qualitative studies, and other non-original research; (ii) non-English and non-Chinese language studies; (iii) studies with irrelevant or unclassifiable exposure data; (iv) duplicate or inaccessible studies. Two authors independently searched all references.

Data Extraction and Processing

Data extraction was performed by two authors, with any disagreements resolved through consensus. The extracted information included the following terms: author, publication year, research year, country, study design and sample size, consumption of nuts, metric and range of AFB1 exposure, RR/OR, 95% CI, etc. [31].

Based on the biomarker type, two approaches were used to estimate daily AFB1 exposure. For urinary AFM1 concentrations (ng/L), daily AFB1 exposure (μg/day) was calculated assuming 1 L urine/day and a conversion factor of 50 [32,33]. For biomarker levels expressed as AFB1-albumin (pg/mg albumin) in serum, values were divided by 100 to estimate the corresponding AFB1 exposure in μg/kg bw/day, assuming a body weight of 60 kg [34]. When adduct levels were reported in fmol/mg, conversion to mass units was performed using the molecular weight of AFB1-lysine (456.1 g/mol), the principal detected by Enzyme-Linked Immunosorbent Assay (ELISA) [35].

Quality Assessment of inclusion studies

**Figure 1:**
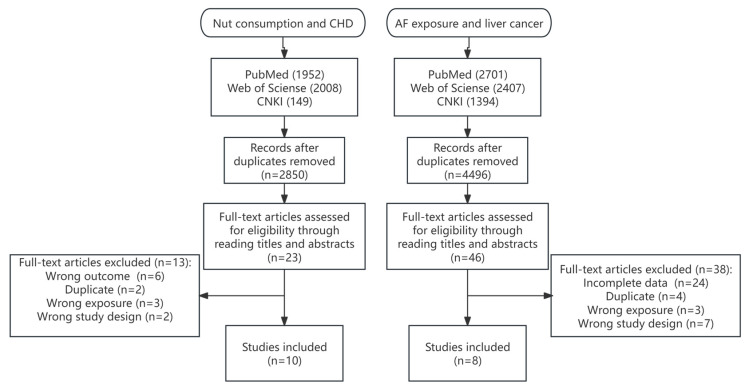
Summary of the study selection process for inclusion.

Study quality was independently assessed by two researchers using the Newcastle–Ottawa Scale (NOS), with scores ≥ 6 indicating high quality [21,36]. Following independent screening by two researchers, 13 cohort studies (10 publications) were included for the analysis of nut consumption and the risk of CHD, involving 1,212,237 participants and 38,365 cases [37,38,39,40,41,42,43,44,45,46]. For AFB1 exposure and the risk of liver cancer, 8 case-control studies [47,48,49,50,51,52,53,54] were included, involving 9450 participants and 2595 cases. The specific search steps and detailed characteristics of the included studies are shown in Figure 1, Table 2 and Table 3, respectively. The NOS scores of all the included studies on the association between nut consumption and the risk of CHD were 6 or higher. In the analysis of AFB1 exposure and liver cancer risk, only the study by Long [48] was excluded, due to an NOS score < 6.

Dose–Response Modeling and Heterogeneity Analysis

The association between nut consumption and the risk of CHD was quantified using RR and 95% CIs, whereas OR and 95% CIs were employed to evaluate the relationship between AFB1 exposure and the risk of liver cancer. Heterogeneity assessment was conducted via Cochran’s Q test and *I^2^* statistics, with thresholds of *I^2^* > 50% and *p* < 0.05 indicating significant heterogeneity [21].

Nonlinear dose–response relationships between nut consumption and CHD were modeled using a two-stage approach, with study-specific estimates fitted via maximum-likelihood methods. A restricted cubic spline (RCS) function with three knots located at the 25th, 50th, and 75th percentiles of the exposure distribution was applied to capture potential nonlinearity [55]. Nonlinearity was tested via the Wald test (*p* < 0.05) [21]. In contrast, a one-stage mixed-effects model with a piecewise linear function was employed to assess the relationship between AFB1 exposure and liver cancer, due to the limited number of available studies and the inclusion of studies with two exposure levels [56]. To account for data sparsity at higher exposure levels of AFB1, the knot was placed at the 25th percentile of the exposure distribution of AFB1.

##### Calculation of DALY

Calculation of the probability of CHD and liver cancer incidence

Following the approach described by Hoekstra, the baseline incidence of CHD and liver cancer in the reference scenario was assumed to represent the average population risk. In the alternative scenarios, relative risks (RRs for CHD and ORs for liver cancer) derived from dose–response relationships were applied to adjust the baseline incidence and estimate the expected disease probabilities [26]:

(2) $$inc=\frac{1}{N}\sum_{i}^{N}\;p_{effect\left(I_{0}\right)}{RR}_{\left(I_{i}\right)}$$

where *inc* is incidence per person; N, the number of individuals in the population; $p_{effect(I_{0})}$ denotes the absolute probability of developing the disease at zero consumption $I_{0}$; and $I_{i}$, the consumption of individual i.

The sex–age prevalence of CHD was obtained from the 2007–2008 China Diabetes and Metabolic Disorders Study [57]. Due to limited data on CHD age distribution in China, this prevalence was assumed to represent the general population. Table S1 presented prevalence, converted incidence, and $p_{effect(I_{0})}$ of CHD in Chinese adults. Moreover, age-stratified liver-cancer incidence rates were obtained from the China Cancer Registry Annual Report (2019) [21]. Table S2 presents the incidence and $p_{effect(I_{0})}$ of liver cancer. Life expectancy was set at 75 years for men and 81 years for women, based on data from China’s National Health Commission.

Calculation of DALYs and net health effect

DALY was the loss of health due to disease. The expected total DALY loss for an individual attributable to a specific disease in the current year can be approximated as [25]:

(3) $$\begin{array}{c} DALY=p_{effect}\;[p_{rec}{YLD}_{rec}W+p_{die}\left({YLD}_{die}W+LE-CA-{YLD}_{die}\right)\\ +\left(1-{p_{die}-p}_{rec}\right)\left(LE-CA\right)W] \end{array}$$

where $p_{effect}$ is the probability of disease onset in the current year (0–1); $p_{rec}$, probability of recovery from the effect (0–1); $p_{die}$, probability this effect causes death (0–1); ${YLD}_{rec}$, duration of disease for those who recover (years); ${YLD}_{die}$, duration of disease (years lived with disease) for those who die of it (years); CA, current age of individual in year of disease onset (years); LE, normal life expectancy (years); and W, disability weight for disease (0–1, where a value of 1 indicated that the health effect was equivalent to death).

In this study, it was assumed that, once the disease occurred, recovery would not be attained during the individual’s lifetime; thus, both $p_{rec}$ and ${YLD}_{rec}$ were assigned a value of 0. Additionally, it was assumed that the probability of disease-related premature mortality was 0; thus, both $p_{die}$ and ${YLD}_{die}$ were set at 0.

The net health impact of the intervention in terms of DALYs was calculated as the following [25]:

(4) $$∆DALY=\sum {DALY}_{alt}-\sum {DALY}_{ref}$$

where $\sum {DALY}_{ref}$ is the aggregate of DALY losses for the reference scenario across all individuals and all health effects; and $\sum {DALY}_{die}$, the aggregate of DALY losses for the alternative scenario across all individuals and all health effects.

### 2.4. Statistical Analyses

All statistical analyses were conducted using *R* statistical software (version 4.4.0). Meta-analytic modeling and graphical visualizations were conducted using metafor and doresmeta packages in *R*. Additionally, *R* was also utilized to fit total AF concentrations and perform statistical analyses of dietary exposure to total AFs from nuts among general adult Chinese consumers.

## 3. Result

### 3.1. Tier 1: Independent Assessment of the Beneficial and Harmful Health Effects of Nut Consumption

#### 3.1.1. The Beneficial Effects of Nut Consumption

Nuts are rich in beneficial nutrients including plant protein, dietary fiber, unsaturated fats, magnesium, tocopherols, tocotrienols, and phenolic compounds. This composition contributes to reduced CHD risk and improved glycemic control [58,59]. Additionally, the fiber and polyphenols support gut health by modulating microbiota, promoting butyrate synthesis, enhancing barrier integrity, and exerting anti-inflammatory effects [60]. Among various outcomes, the evidence supporting the protective effect of nuts against CHD is the most robust. Several prospective cohort studies have demonstrated that higher nut consumption is associated with a reduced risk of CHD [43,61]. A meta-analysis revealed that high nut consumption was inversely associated with CHD (RR 0.82; 95% CI 0.76–0.89) compared to low nut consumption [55]. Based on the strength, consistency, and quality of the available evidence, the association between nut consumption and reduced CHD risk has been graded as “convincing”. In contrast, meta-analyses on nut consumption benefits for diabetes and anti-inflammatory effects report non-significant associations, with evidence levels below “convincing” [62,63,64].

#### 3.1.2. Risks Associated with Nut Consumption

Nuts may be contaminated with AFs, a Group 1 carcinogen, leading to potential human exposure [10]. A cohort study conducted in Shanghai reported that AF exposure was associated with a relative risk of 3.5 (95% CI: 1.5–8) for liver cancer [33]. Moreover, the hepatitis B virus and AF exposure exhibit synergistic effects in the development of HCC, indicating that the risk of HCC is markedly elevated when both factors co-occur [65]. The WHO has categorized the level of evidence for AF exposure and liver cancer as “convincing” [66]. While animal and in vitro studies demonstrated AFs’ immunotoxicity [67,68], meta-analytical evidence remains limited, resulting in a “possible” evidence classification for this effect.

In Tier 1, only the beneficial effect of nut consumption on CHD and the adverse effect of AF exposure on liver cancer were supported by a “convincing” level of evidence. Consequently, only these two health outcomes were selected for further evaluation in Tier 2.

### 3.2. Tier 2: Qualitative Assessment of the Risks and Benefits Associated with Nut Consumption

Based on the Global Burden of Disease (GBD) data, Table 4 presents the severity and population impact of selected health outcomes, including CHD and liver cancer. Age-standardized DALY rates were 2132.1 per 100,000 person-years for CHD and 253.6 per 100,000 person-years for liver cancer [69]. Age-standardized mortality rates were 108.7 per 100,000 person-years for CHD [70] and 10.2 per 100,000 person-years for liver cancer [71]. The severity of health effects, measured by disability weight, was 0.790 for CHD and 0.857 for liver cancer [72]. Given the considerable uncertainty in the qualitative assessment, a more precise quantitative assessment was required. Therefore, the assessment proceeded to tier 3.

### 3.3. Tier 3 and Tier 4

#### 3.3.1. Dose–Response Relationship

**Figure 2:**
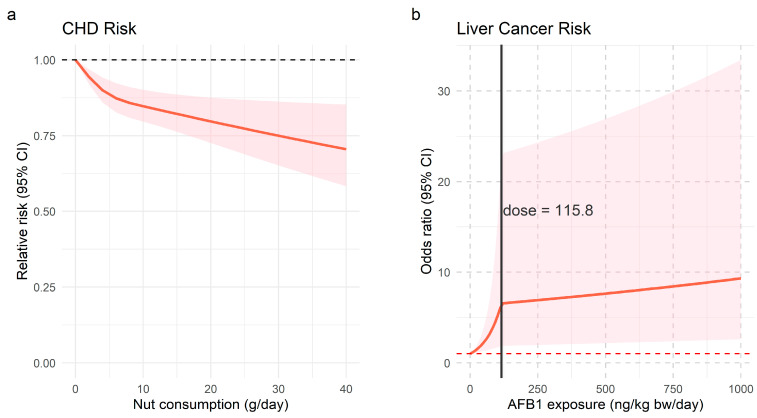
Dose–response relationships between dietary exposures and disease risks. (**a**) Dose-response relationship between nut consumption and CHD risk; (**b**) Dose-response relationship between AFB1 exposure and liver cancer risk.

In this study, higher consumption was associated with a significantly reduced risk of CHD (RR= 0.81, 95% CI: 0.72–0.91), with high heterogeneity (*I^2^* = 85.39%, *p* < 0.0001). Sensitivity analysis, excluding Guasch-Ferré et al. [40], slightly weakened the association (RR = 0.85, 95% CI 0.78–0.92), with reduced heterogeneity (*I*^2^ = 59.34%, *p* = 0.001). Subgroup analyses (shown in Table 5) revealed no significant differences by region, follow-up duration, or adjustment for hypertension. The dose–response analysis revealed a nonlinear relationship, indicating that at a nut consumption level of 30 g/day, the risk of CHD was RR = 0.75 (95% CI: 0.65–0.86) (Figure 2).

**Figure 3:**
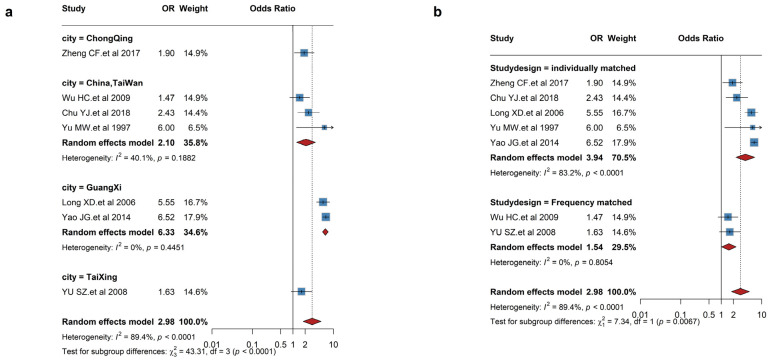
Stratified forest plots showing the association between AFB1 exposure and liver cancer (**a**) by region and (**b**) by study design [47,49,50,51,52,53,54].

Similarly, the high exposure level of AFB1 was associated with an increased risk of liver cancer (RR = 2.94, 95% CI: 1.28–6.78), with high heterogeneity (*I*^2^ = 89.17%, *p* < 0.0001). Exclusion of the studies by Yao et al. [52] and Long et al. [49] on sensitivity analysis resulted in a markedly lower heterogeneity, with an RR of 1.87 (95% CI: 1.41–2.51, *I*^2^ = 0%, *p* = 0.46). Subgroup analysis indicated significant differences by region and study design (Figure 3). A piecewise linear dose–response relationship was observed, with an OR of 1.02 (95% CI: 1.01–1.03) for liver cancer at 1 ng/kg bw/day AFB1 exposure (Figure 2).

#### 3.3.2. DALY

**Figure 4:**
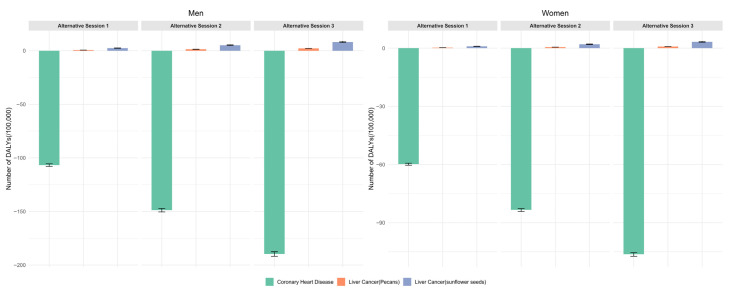
Change in DALYs by sex as the reference scenarios transfer to alternative scenarios.

Figure 4 illustrates the changes in DALYs among men and women under three alternative scenarios of nut consumption (10, 20, and 30 g/day). Overall, compared to the current average nut consumption of 4.66 g/day, DALYs saved from reduced CHD burden increased with higher consumption, while DALYs lost due to elevated AFs-related liver cancer risk also rose slightly. The net benefit remained positive across all scenarios, with men experiencing greater health gains than women. As shown in Table 6, compared to the reference scenario, increasing nut consumption to 10, 20, and 30 g/day resulted in DALY reductions of 104.39, 143.63, and 181.47 for men, and 58.79, 81.29, and 102.94 for women, respectively, under the sunflower seed scenario. Corresponding reductions under the pecan scenario were 106.08, 147.40, and 187.56 for men, and 59.47, 82.81, and 105.41 for women.

### 3.4. Uncertainty Analysis

Some uncertainties may have influenced the results in this study. First, the dose–response relationship between nut consumption and the risk of CHD was derived mainly from international studies, which may not fully capture the health status and dietary characteristics of the Chinese population. Meanwhile, AFB1 exposure estimates were based on biomarker conversions using literature-derived parameters, introducing further uncertainty. Secondly, the concentrations of AFB1, AFB2, AFG1, AFG2 in nuts were based on China National Food Safety Monitoring data in 2013. The concentration of AFs in nuts varied with region, time, temperature, and humidity. In addition, due to limited data on compound-specific carcinogenic potency, the carcinogenic potencies of AFB2, AFG1, and AFG2 were assumed to be equivalent to that of AFB1, which may have led to a slight overestimation of the total risk from AF exposure. Thirdly, the use of non-local disability weights in DALY calculations may have led to estimation bias. Finally, it was assumed that the average incidence within an age group represented the entire group, which may have overlooked intra-group variability and led to biased or imprecise estimations.

## 4. Discussion

Despite existing studies on the cardiovascular benefits of nut consumption and the potential health risks associated with AF exposure, comprehensive assessments that integrate both the benefits and risks remain limited. This study applied the BRAFO framework to quantitatively assess the health impact of nut consumption among Chinese adults. Compared with the current consumption of nuts, a daily consumption of 30 g of nuts yielded the greatest net health benefits, particularly among men. Sunflower seeds and pecans were selected as representative nuts in alternative scenarios to capture the potential range of outcomes, given their high and low AF contamination levels, respectively. The resulting DALY estimates reflected the upper and lower bounds of health impact. Based on this comparison, it is advisable for consumers to prioritize nuts with lower aflatoxin contamination, such as pecans and chestnuts, to minimize risk.

Furthermore, the simple distribution models were employed to estimate the dietary exposure to AFB1 and total AFs across the different consumption scenarios. Given the carcinogenic and genotoxic potential of AFs, the margin of exposure (MOE) approach was applied to assess the associated human health risks. The benchmark-dose lower confidence limit 10% (BMDL_10_) of 0.4 μg/kg bw/day was selected as the reference point [29]. MOE was calculated as the ratio of BMDL10 to estimated dietary AFB1 exposure, with values above 10,000 indicating low concern and those below 10,000 suggesting potential risk requiring attention. The results indicated that, at a nut consumption of 10 g/day, the mean and 90th percentile (P90) MOE values for AFB1 were above 10,000 (17,740 and 15,020, respectively), while those for total AFs were below 10,000 (4393 and 3719, respectively), based on LB estimates (Table 7). At nut consumption levels of 20 g/day and 30 g/day, the P90 MOE for both AFB1 and total AFs were less than 10,000, indicating a potential health concern. Although AFB1 and AFG1 were classified as carcinogenic, evidence for AFB2 and AFG2 was limited or inadequate [73]. Notably, AFB1 was considered more potent than AFG1 [74]. In this study, the potency factors of AFB2, AFG1, and AFG2 were assumed to be equivalent to that of AFB1, which may have led to an overestimation of the risk associated with AF exposure. Therefore, despite the greater health gains observed at 30 g/day, a daily intake of 10 g is recommended to balance benefit and safety.

Eneroth et al. [75] also conducted a quantitative RBA, and observed health gains with increased nut consumption from 5 g/day to 30 g/day among Swedish adults aged 55–79 years, considering both CVD benefits (MI and stroke reduction) and AFB1-related liver cancer risk. However, their analysis focused only on AFB1 and relied on literature-based dose–response estimates. This study incorporated four major AFs (AFB1, AFB2, AFG1, and AFG2) and applied a dose–response meta-analysis, enabling a more comprehensive exposure assessment and evidence-based risk estimation. Additionally, while Eneroth’s study estimated DALYs saved from nut consumption based on MI and stroke, this study focused exclusively on CHD, as current evidence linking nut consumption with stroke risk remains inconclusive. A prospective urban–rural epidemiological study encompassing 16 nations across 5 continents revealed no significant correlation between nut consumption and stroke incidence (mvHR: 0.98; 95% CI: 0.84, 1.14; *p* = 0.76) [43]. Some meta-analyses also indicated that heightened nut consumption did not correlate with stroke incidence [55,76,77]. Consequently, due to the limited and inconclusive meta-analytic evidence on the relationship between nut consumption and stroke prevention, coupled with substantial uncertainty, this study concentrated solely on exploring the dose–response association between nut consumption and CHD risk reduction.

The RBA approach has been widely applied to evaluate the health impacts of various foods, such as fish [14], thermally processed foods [17], and coffee [18]. Carvalho et al. [14] also focused on CVD outcomes, applying an RBA framework to assess the net health impact of fish and seafood consumption scenarios in the Portuguese population. While their study categorized exposure into quartiles and estimated RR based on the median intake of each group, the present study applied a dose–response meta-analysis to derive RR across the continuous intake range. This approach provides a more precise characterization of the exposure–response relationship, offering a refined basis for RBA that better reflects the continuous nature of dietary exposure.

In this study, we further explored the dose–response relationship between nut consumption and CHD risk using a RCS model, which allowed for the assessment of potential non-linear associations. The meta-analysis findings regarding nut consumption and CHD risk were consistent with those from several international studies [1,55]. In Arnesen’s study, a one-stage mixed-effects model was employed to assess the dose–response relationship between nut consumption and CHD risk. However, specific model coefficients were not reported, rendering it difficult to directly apply their findings to the present analysis. In this study, a two-stage dose–response approach was adopted. Although fewer publications were included compared to Arnesen’s analysis, the final results were consistent between the two approaches; notably, both studies reported an RR of 0.75 at a nut consumption level of 30 g/day. In addition to the analysis of CHD risk, this study also addressed the dose–response relationship between AFB1 exposure and liver cancer risk. This study was the first meta-analysis to establish a dose–response relationship between AFB1 exposure and liver cancer risk using data from the Chinese population. A one-stage mixed-effects model was applied to conduct the dose–response meta-analysis, enabling the inclusion of a broader range of studies. He et al. [21] also investigated this relationship based on a single prospective cohort study, and established a linear dose–response model. However, by employing a dose–response meta-analysis approach, this study was able to synthesize evidence from multiple studies to define the overall association and explore between-study heterogeneity [78]. Compared with individual studies, meta-analysis can provide more precise estimates than individual studies, minimizing bias and reducing random error [79].

The AF concentration data used in this study were derived from the China National Food Safety Monitoring data in 2013. Over the past decade, regulatory frameworks and industry practices for AF control in China have been continuously improved [80]. Feed and food enterprises have progressively strengthened standardized management protocols, while governmental authorities have refined legislation and supervision across production, consumption, and distribution chains [80]. In particular, GB 31653-2021 established comprehensive process-based requirements for AF control in nuts such as peanuts, walnuts, and pine nuts, covering harvesting, drying, storage, transportation, and processing [81]. Despite the fact that the 2013 data may have a temporal limitation, the data still offer a nationally representative and reliable basis for exposure assessment and risk estimation. While more recent studies reported AF concentrations for specific nut types, such as chestnuts in Shandong Province [82], peanuts in Zhejiang Province [83] and peanuts, sunflower seeds, and pine nuts in Chongqing Province [84], our dataset provided a uniquely broad coverage of both geographic regions and nut varieties. In some cases, AF concentrations reported in these studies were higher than those observed in the 2013 nationwide monitoring data (e.g., chestnuts in Shandong [82]), indicating that certain nuts may have an increasing contamination trend. Considering this, continued monitoring and expanded assessment remain necessary.

## 5. Conclusions

This study applied the BRAFO framework to conduct a quantitative RBA of increased nut consumption among Chinese adults, incorporating nationally representative dietary data and contaminant surveillance results. Through dose–response meta-analyses, quantitative relationships were established between nut consumption and the risk of CHD, as well as between AFB1 exposure and liver cancer. A Monte Carlo simulation was applied to account for variability and uncertainty, with disease burden changes estimated in DALYs. Results showed that increased nut consumption, particularly at 30 g/day, significantly reduced DALYs, primarily due to cardiovascular benefits. Although AF exposure and liver cancer risk rose slightly with higher intake, net health outcomes remained positive across all scenarios. By modeling two representative nut types, sunflower seeds with relatively high AF contamination and pecans with low contamination, the analysis confirmed that benefits outweighed risks, even under conservative assumptions. Taking into account the exposure assessment and overall risk–benefit analysis, a daily intake of 10 g of nuts is recommended. These findings support promoting nut consumption in China while highlighting the need for continued AF control to optimize health benefits.

## Figures and Tables

**Table 1:** Parameter fitting concentrations of total AFs in nut samples.

Nuts	Number	Positive Samples (%)	Mean (μg/kg)		P95 (μg/kg)		Max (μg/kg)	
			LB ^a^	UB ^a^	LB ^a^	UB ^a^	LB ^a^	UB ^a^
Ginkgo nut	19	4 (21.05)	0.31	0.67	1.32	1.41	4.00	4.00
Walnut	171	14 (8.19)	0.12	0.51	0.12	0.52	6.70	7.10
Chestnut	9	0 (0.00)	0.00	0.40	0.00	0.40	0.00	0.40
Pine nut	206	25 (10.68)	0.41	0.78	1.38	1.43	41.02	41.12
Almond	413	54 (13.08)	0.20	0.58	1.00	1.26	11.20	11.50
Cashew	62	5 (8.06)	0.10	0.49	0.93	1.22	1.94	2.24
Hazelnut	38	1 (2.63)	0.03	0.42	0.00	0.40	0.98	1.08
Pistachio	490	56 (11.43)	0.16	0.54	0.81	1.04	18.00	18.25
Peanut	91	5 (5.49)	0.16	0.54	0.40	0.75	6.90	7.20
Sunflower seed	896	102 (11.38)	1.05	1.44	2.00	2.30	82.37	82.42
Pumpkin seed	357	37 (10.36)	0.28	0.66	0.84	1.12	31.00	31.10
Watermelon seed	419	38 (9.07)	0.10	0.48	0.69	0.99	3.96	4.21
*Amygdalus communis* L.	92	6 (6.52)	0.22	0.61	0.50	0.78	9.79	9.84
Macadamia nut	16	2 (12.50)	0.21	0.57	1.66	1.68	1.83	1.93
Pecan	48	0 (0.00)	0.00	0.40	0.00	0.40	0.00	0.40
Other ^b^	22	2 (9.09)	0.19	0.57	0.08	0.48	4.00	4.00
Total	3349	348 (10.39)	0.42	0.80	0.99	1.21	82.37	82.42

^a^: LB: lower bound, UB: upper bound. ^b^: nuts of unclassified types.

**Table 2:** Characteristics of studies included in nut consumption and risk of CHD dose–response meta-analysis.

Author, Year	Average Follow-Up Time (Year)	Country	Outcome	Sample Size (Cases)	Dose	RR	NOS	Adjustment Factor
Fraser et al. (1992) [37]	5	USA	Fatal CHD	31,208 (260)	<1 serving/week	1	7	Age, sex, smoking, exercise, relative weight, high blood pressure and all food variables.
					1–4 servings/week	0.76 (0.56, 1.04)		
					≥5 servings/week	0.52 (0.36, 0.76)		
			Nonfatal MI	31,208 (134)	<1 serving/week	1		
					1–4 servings/week	0.78 (0.51, 1.18)		
					≥5 servings/week	0.49 (0.28, 0.85)		
Albert et al. (2002) [38]	17	USA	Nonfatal MI	22,071 (1037)	1 serving/month	1	7	Age (continuous), aspirin and beta carotene treatment assignment, evidence of cardiovascular disease before 12-month questionnaire, BMI, smoking, history of diabetes, history of hypertension, history of hypercholesterolemia, alcohol consumption, vigorous exercise, vitamin E, vitamin G and multivitamin use at baseline.
					1–3 servings/month	1.22 (1, 1.51)		
					1 serving/week	1.20 (0.96, 1.50)		
					≥2 servings/week	1.04 (0.82, 1.33)		
							
Haring et al. (2014) [39]	22	USA	CHD	12,066 (1147)	0 serving/day	1	8	Age, sex, race, study center, total energy intake, smoking, education, systolic blood pressure, use of antihypertensive medication, HDLc total cholesterol, use of lipid-lowering medication, body mass index, waist-to-hip ratio, alcohol intake, sports-related physical activity, leisure-related physical activity, carbohydrate intake, fiber intake, and magnesium intake.
					0.1 serving/day	0.89 (0.75, 1.06)		
					0.2 serving/day	0.86 (0.71, 1.05)		
					0.4 serving/day	0.83 (0.68, 1.01)		
					1.0 serving/day	0.91 (0.74, 1.12)		
							
Guasch-Ferré et al. (2017) [40]	32	USA	MI	76,364 (3552)	0 g/day	1	7	Age, Caucasian, BMI, physical activity, smoking status, physical examination for screening purposes, current multivitamin use, current aspirin use, family history of diabetes mellitus, myocardial infarction or cancer, history of diabetes mellitus, hypertension or hypercholesterolemia, intake of total energy, alcohol, red or processed meat, fruits and vegetables, and, in women, menopausal status and hormone use. In the NHS II study, the multivariable model was further adjusted for oral contraceptive use.
					1.68 g/day	0.84 (0.76, 0.91)		
					3.92 g/day	0.76 (0.68, 0.86)		
					9.24 g/day	0.73 (0.64, 0.82)		
					27 g/day	0.69 (0.56, 0.83)		
				92,946 (670)	0 g/day	1		
					1.47 g/day	0.93 (0.77, 1.13)		
					3.92 g/day	0.79 (0.61, 1.02)		
					7.98 g/day	0.84 (0.63, 1.12)		
					23.03 g/day	0.57 (0.27, 1.23)		
				41,526 (4168)	0 g/day	1		
					1.96 g/day	0.93 (0.84, 1.03)		
					3.92 g/day	0.90 (0.81, 0.99)		
					7.84 g/day	0.88 (0.79, 0.97)		
					24.08 g/day	0.86 (0.76, 0.98)		
Larsson et al. (2018) [41]	17	Sweden	MI	61,364 (4983)	0	1	8	Education, family history of myocardial infarction before 60 years of age, smoking, walking/bicycling, exercise, aspirin use and consumption of alcohol, fruits, vegetables and total energy, potential intermediates of the nut-CVD relationship, including body mass index, history of diabetes, history of hypertension and history of hypercholesterolemia.
					1–3 servings/month	0.98 (0.92, 1.04)		
					1 serving/week	0.91 (0.79, 1.05)		
					≥3 servings/week	0.88 (0.70, 1.11)		
							
Perez-Cornago et al. (2020) [42]	12.6	Multinational		490,311 (8504)	0	1	7	Age, smoking status and number of cigarettes per day, histories of diabetes, hypertension and hyperlipidemia, including Cambridge physical activity index, employment status, level of education completed, current consumption, BMI, and observed intakes of total energy, red and processed meat, and cheese are considered in the analysis.
					0.006–0.5 g/day	0.99 (0.90, 1.08)		
					0.5–2.0 g/day	0.97 (0.91, 1.04)		
					2.0–5.3 g/day	0.96 (0.89, 1.03)		
					>5.3 g/day	0.93 (0.86, 1.01)		
							
de Souza et al. (2020) [43]	9.5	Multinational	MI	124,329 (2559)	<30 g/month	1	8	Age, sex, location, and center, follow-up time, lifestyle factors such as education, tobacco use, BMI, waist-to-hip ratio, physical activity, family history of CVD, diabetes, and cancer, and diet factors including fish, fruits, vegetables, red/processed meat, legumes, and total energy.
					30 g/month–30 g/week	0.97 (0.85, 1.12)		
					30 g/week–120 g/week	0.99 (0.87, 1.13)		
					≥120 g/week	0.86 (0.72, 1.04)		
Ivey et al. (2021) [44]	3.5	USA	CAD	179,827 (9908)	<1 serving/month	1	7	Age, sex, race, BMI, smoking status, alcohol intake, physical activity, education, modified DASH score.
					1–3 servings/month	0.93 (0.89, 0.99)		
					1 servings/week	0.89 (0.84, 0.95)		
					2–4 servings/week	0.83 (0.78, 0.89)		
					≥5 servings/week	0.78 (0.72, 0.84)		
Mohammadifard et al. (2021) [45]	11.25	Iran	IHD	5432 (594)	0.64 g/day	1	7	Age, sex, education, residence area, smoking status, daily physical activity, family history of CVD, diabetes mellitus, hypertension, hypercholesterolemia and aspirin use, and menopausal status in female, BMI and dietary factors.
					0.66 g/day	1.07 (0.84, 1.37)		
					0.96 g/day	1.01 (0.79, 1.28)		
					2.28 g/day	0.98 (0.76, 1.27)		
							
Ikehara et al. (2021) [46]	14.8	Japan	IHD	74,793 (849)	0 g/day	1	7	Age, sex, PHC, smoking status, alcohol consumption, perceived stress level, physical activity, and vegetable, fruit, fish, soy, sodium, and total energy intakes, BMI, history of hypertension, history of diabetes, and cholesterol-lowering drug.
					0.7 g/day	0.98 (0.79, 1.21)		
					1.3 g/day	0.93 (0.77, 1.11)		
					4.3 g/day	0.97 (0.80, 1.17)		
							

Abbreviations: BMI: Body Mass Index; CAD: Coronary Artery Disease; CHD: Coronary Heart Disease; CVD: Cardiovascular Diseases; DASH: Dietary Approaches to Stop Hypertension; HDL: High-Density Lipoprotein; IHD: Ischemic Heart Disease; MI: Myocardial Infarction; PHC: Public Health Center.

**Table 3:** Characteristics of studies included in AFB1 exposure and risk of liver cancer dose–response meta-analysis.

Author, Year	Country	Research Year	Study Design	Matching Factors	Biomarker	Dose	Case	Control	OR	NOS	Adjustment Factor
Yu MW et al. (1997) [47]	China, Taiwan	1988–1992	Individually matched	Age, interview, urine collection time.	urinary AFM1 (ng/mL)	<1.61	9	18	1	7	Educational level, ethnicity, habitual alcohol drinking, and cigarette smoking status.
						1.61–2.85	10	10	1.9 (0.5, 7.2)		
						>2.85	23	15	6 (1.2, 29)		
Long XD et al. (2005) [48]	GuangXi	-	Frequency matched	Age, sex, ethnicity.	AFB1 (μg/day)	>7	536	447	1	4	-
						<7	140	71	5.82 (3.26, 10.38)		
Long XD et al (2006) [49]	GuangXi	2004–2005	Individually matched	Age, sex, ethnicity, and hepatitis B virus infection.	AFB1 (μg/day)	>7	58	127	1	6	Adjusted for age, sex, ethnicity, HBV infection, anti-HCV, and AFB1 exposure levels.
						<7	175	130	5.55 (3.82, 8.06)		
									
YU SZ et al. (2008) [50]	TaiXing	2000	Frequency matched	Age, length of residence.	AFB1 albumin adduct (fmol/mg)	<247	33	94	1	8	Age, gender, BMI, education, alcohol consumption, smoking, virus infection.
						247.1–388.9	46	94	1.15 (0.61, 2.14)		
						388.9–545	42	95	1.19 (0.61, 2.21)		
						>545.1	61	94	1.63 (0.9, 2.96)		
Wu HC et al. (2009) [51]	China, Taiwan	1990–2004	Frequency matched	Age, gender, residential township, recruitment date.	AFB1-albumin adducts (fmol/mg)	<26.9	66	263	1	8	Aflatoxin biomarker assay batch, HBsAg, anti-HCV status, habitual smoking, alcohol consumption, and BMI.
						26.9–43.5	58	262	1.11 (0.69, 1.83)		
						43.5–71.35	49	264	1.18 (0.69, 2.03)		
						>71.35	57	263	1.47 (0.83, 2.58)		
										
Yao JG et al. (2014) [52]	GuangXi	2004–2012	Individually matched	Age, ethnicity, sex, HBV and HCV infection.	AFB1-albumin adducts (ln fmol/mg)	<2.18	352	1060	1	7	-
						2.18–2.98	417	604	2.08 (1.75, 2.47)		
						>2.98	717	332	6.52 (5.46, 7.79)		
Zheng CF et al. (2017) [53]	ChongQing	2013–2016	Individually matched	Age, gender.	AFB1-albumin adducts (ng/g)	<133.1	81	132	1	7	Smoking, drinking alcohol, HBV infection, family history of HBV infection, family history of tumors, diabetes, and hyperglycemia.
						≥133.1	133	82	1.9 (1.1, 3.4)		
									
										
Chu YJ et al. (2018) [54]	China, Taiwan	1991–2011	Individually matched	Age, gender, residence, date of blood sample collection.	AFB1-albumin adducts (fmol/mg)	<21.5	23	1475	1	9	Age, sex, education level, smoking status, drinking status, HBsAg status.
						>21.5	21	468	2.43 (1.31, 4.52)		
									
									

**Table 4:** Different dimensions of health effects.

Health Impact Endpoint	Incidence of Health Effects	Severity of Health Effects [72]	Effect Duration	Age-Standardized DALY (1/105) [69]	Age-Standardized Mortality (1/105) (95% CI)	Changes in Health Effects
Coronary heart disease	Decrease	0.790	Lifelong after illness	2132.1 (2093.7, 2179.8) ^a^	108.7 (99.8, 115.6) ^a^ [70]	Beneficial
Liver cancer	Increase	0.857	Lifelong after illness	253.6 (243.2, 266.2)	10.2 (9.8, 10.7) [71]	Adverse

a: The data were replaced by data on ischemic heart disease.

**Table 5:** Subgroup analysis of high vs. low nut consumption and risk of CHD.

Subgroup	N Studies	OR (95% CI)	*p* for Group Differences
Region			0.076
Europe	1	0.88(0.70, 1.1)	
USA	8	0.74 (0.62, 0.87)	
Asia	2	0.97 (0.84, 1.13)	
Multinational	2	0.92 (0.85, 0.99)	
Follow-up time			0.18
<10 years	4	0.77 (0.69, 0.86)	
≥10 years	9	0.82 (0.76, 0.90)	
Adjusted for hypertension			0.72
NO	3	0.82 (0.75, 0.90)	
YES	10	0.79 (0.68, 0.92)	
Overall	13	0.81 (072, 0.91)	

**Table 6:** Mean DALYs and mean change in DALYs resulting nut consumption for different scenarios in adults (1/100, 000).

Scenarios ^c^	Man			Woman		
	CHD	Liver Cancer	ΔDALY	CHD	Liver Cancer	ΔDALY
	sunflower seeds ^a^					
reference scenario	810.08 (802.71, 818.68)	801.04 (797.97, 803.97)		452.47 (448.56, 456.32)	269.82 (268.77, 271.15)	
alternative scenario 1	703.34 (697.08, 710.90)	803.39 (800.37, 806.25)	−104.39 (−105.51, −103.24)	392.73 (389.22, 398.04)	270.08 (269.03, 271.41)	−58.79 (−59.33, −58.38)
alternative scenario 2	661.33 (655.44, 668.45)	806.16 (803.07, 809.17)	−143.63 (−145.17, −142.03)	369.12 (365.78, 372.24)	270.26 (269.30, 271.69)	−81.29 (−82.00, −80.65)
alternative scenario 3	620.45 (614.92, 627.15)	809.20 (806.00, 811.93)	−181.47 (−183.80, −179.64)	346.23 (234.09, 349.16)	270.64 (269.59, 271.98)	−102.94 (−103.83, −102.17)
	pecans ^b^					
reference scenario	810.08 (802.71, 818.68)	801.04 (797.97, 803.97)		452.47 (448.56, 456.32)	269.82 (268.77, 268.77)	
alternative scenario 1	703.34 (697.08, 710.90)	801.70 (798.63, 804.63)	−106.08 (−107.26, −104.89)	392.73 (389.22, 398.04)	270.76 (269.70, 272.12)	−59.47 (−60.03, −59.03)
alternative scenario 2	661.33 (655.44, 668.45)	802.39 (799.32, 805.33)	−147.40 (−149.05, −145.77)	369.12 (365.78, 372.24)	271.88 (270.89, 273.16)	−82.81 (−83.59, −82.19)
alternative scenario 3	620.45 (614.92, 627.15)	803.11 (800.04, 806.05)	−187.56 (−189.65, −185.56)	346.23 (234.09, 349.16)	273.11 (272.06, 274.49)	−105.41 (−106.38, −104.62)

^a^: Increase consumption of sunflower seeds in alternative scenarios. ^b^: Increase consumption of pecans in alternative scenarios. ^c^: Reference scenario indicates current nut consumption; alternative scenarios 1, 2, and 3 correspond to daily consumption of 10 g, 20 g, and 30 g, respectively.

**Table 7:** Estimated MOE values of AFB1 and total AF exposure from nuts in different scenarios among Chinese adults.

Scenarios ^a^	Descriptive Level	MOE ^b^ of AFB1		MOE ^b^ of Total AFs	
		LB	UB	LB	UB
Reference scenario					
	p50	N/A ^c^	N/A ^c^	N/A ^c^	N/A ^c^
	p75	N/A ^c^	N/A ^c^	N/A ^c^	N/A ^c^
	p90	**15,751** ^d^	5648	3900	2035
	mean	**50,553** ^d^	**18,127** ^d^	**12,519** ^d^	6532
Alternative scenario 1					
	p50	**23,108** ^d^	8286	5722	2986
	p75	**20,027** ^d^	7181	4959	2588
	p90	**15,020** ^d^	5386	3719	1941
	mean	**17,740** ^d^	6361	4393	2292
Alternative scenario 2					
	p50	**11,554** ^d^	4143	2861	1493
	p75	**10,398** ^d^	3729	2575	1344
	p90	8858	3176	2194	1145
	mean	**10,546** ^d^	3781	2611	1363
Alternative scenario 3					
	p50	7728	2771	1914	999
	p75	7048	2527	1745	911
	p90	6162	2210	1526	796
	mean	7422	2661	1838	959

^a^: Reference scenario indicates current nut consumption; alternative scenarios 1, 2, and 3 correspond to daily consumption of 10 g, 20 g, and 30 g, respectively. ^b^: MOE: Margin of Exposure. ^c^: Not Available: MOE values cannot be calculated because the AFB1 or total AF exposure was 0 μg/kg bw/day. ^d^: Margin of exposures with low public health concern (>10,000) are bolded.

## Data Availability

The original contributions presented in this study are included in the article/supplementary material. Further inquiries can be directed to the corresponding authors.

## Supplementary Materials

The following supporting information can be downloaded at: https://www.mdpi.com/article/10.3390/foods14203498/s1, Table S1: The prevalence, incidence and $p_{effect(I_{0})}$ of CHD by sex and age group; Table S2: The incidence and $p_{effect(I_{0})}$ of liver cancer by sex and age group. References [21,57] are cited in the supplementary materials.

## Abbreviations

Abbreviation	Definition
AF	Aflatoxin
AFB1	Aflatoxin B1
AFB2	Aflatoxin B2
AFG1	Aflatoxin G1
AFG2	Aflatoxin G2
BMDL10	Benchmark Dose Lower Confidence Limit 10%
BMI	Body Mass Index
BRAFO	Benefit–Risk Analysis for Foods
CAD	Coronary Artery Disease
CHD	Coronary Heart Disease
CI	Confidence Interval
CVD	Cardiovascular Diseases
DALY	Disability-Adjusted Life Year
DASH	Dietary Approaches to Stop Hypertension
ELISA	Enzyme-Linked Immunosorbent Assay
GBD	Global Burden of Disease
HBV	Hepatitis B Virus
HCC	Hepatocellular Carcinoma
HDL	High-Density Lipoprotein
HPLC	High-Performance Liquid Chromatography
IARC	International Agency for Research on Cancer
IHD	Ischemic Heart Disease
LOD	Limits of Detection
MI	Myocardial Infarction
MOE	Margin of Exposure
NOS	Newcastle–Ottawa Scale
OR	Odds Ratio
PHC	Primary Health Care
RBA	Risk–Benefit Assessment
RCS	Restricted Cubic Spline
RR	Relative Risk
