# Supplementary material for: Application of BRAFO-Tiered Approach for Risk–Benefit Assessment of Nut Consumption in Chinese Adults
Source: Foods. 2025 Oct 14;14(20):3498. doi: 10.3390/foods14203498 (PMC12564558; doi:10.3390/foods14203498)
Supplement: Supplementary file 1 [file foods-14-03498-s001.zip › foods-3907928-supplementary.pdf]

Supplementary Material

Table S1. The prevalence, incidence and $p_{effect(I_0)}$ of CHD by sex and age group

Age group	men			women		
	prevalence [1]	incidence	$p_{effect(I_0)}$	prevalence [1]	incidence	$p_{effect(I_0)}$
18-29	0	0.00	0.00	10	0.18	0.19
30-39	80	2.00	2.08	30	0.54	0.56
40-49	410	13.67	14.32	160	4.44	4.69
50-59	1370	68.50	71.88	920	35.38	37.20
60-69	1850	185.00	194.24	1030	64.38	67.70
70+	2340	2340.00	2461.22	2460	410.00	427.82

Table S2. The incidence and $P_{effect(I_0)}$ of liver cancer by sex and age group

Age_group	men		women	
	incidence [2]	$P_{effect(I_0)}$	incidence [2]	$P_{effect(I_0)}$
18-19	0.43	0.43	0.19	0.19
20-24	0.83	0.83	0.42	0.42
25-29	2.59	2.59	0.67	0.67
30-34	6.39	6.39	1.42	1.42
35-39	13.60	13.59	2.62	2.61
40-44	28.38	28.36	5.61	5.60
45-49	45.67	45.58	9.83	9.81
50-54	64.37	64.32	14.42	14.41
55-59	86.68	86.61	22.24	22.22
60-64	109.55	109.47	34.47	34.44
65-69	124.13	124.04	47.89	47.85
70-74	130.20	130.11	61.75	61.71
75-79	143.96	143.86	81.77	81.73
80-84	161.79	161.71	94.00	93.96
85+	151.16	151.06	86.97	86.96

1. Yang, Z.; Liu, J.; Ge, J.; Chen, L.; Zhao, Z.; Yang, W.; China National Diabetes and Metabolic Disorders Study Group Prevalence of Cardiovascular Disease Risk Factor in the Chinese Population: The 2007–2008 China National Diabetes and Metabolic Disorders Study. *Eur. Heart J.* **2012**, *33*, 213–220, doi:10.1093/eurheartj/ehr205.
2. He, M.; Lyu, X. Application of BRAFO-Tiered Approach for Health Benefit-Risk Assessment of Dark Tea Consumption in China. *Food Chem. Toxicol.* **2021**, *158*, 112615, doi:10.1016/j.fct.2021.112615.
